# *BMC Women’s Health* reviewer acknowledgement 2015

**DOI:** 10.1186/s12905-016-0289-3

**Published:** 2016-02-15

**Authors:** Nawsheen Boodhun

**Affiliations:** BioMed Central, Floor 6, 236 Gray’s Inn Road, London, WC1X 8HB UK

## Abstract

The editors of *BMC Women’s Health* would like to thank all our reviewers who have contributed to the journal in Volume 15 (2015).

**Julie Abayomi**

UK

**Misra Abdulahi**

Ethiopia

**Gedefaw Abeje**

Ethiopia

**Philip Adongo**

Ghana

**Kelvin Afrashtehfar**

Canada

**Maryam Ahmadian**

Malaysia

**Leonard Ajah**

Nigeria

**Tomi Akinyemiju**

USA

**Angela Akol**

Uganda

**Melissa Aldrich**

USA

**Habiba Isse Ali**

United Arab Emirates

**Nisreen Alwan**

UK

**Eyitope Amu**

Nigeria

**Sari Andajani**

New Zealand

**Troy Andersen**

USA

**Eleanor Anderson**

USA

**Ewa Andersson**

Sweden

**Muhammad Arif**

USA

**Ludwin Artur**

Poland

**Ian Askew**

Kenya

**Nega Assefa**

Ethiopia

**Lou Atkinson**

UK

**Jodie Avery**

Australia

**Susan Ayers**

UK

**Shawky Badawy**

USA

**Donna Baird**

USA

**Ana Paula Barbosa**

Portugal

**Lesley Barclay**

Australia

**Janine Barden-O'Fallon**

USA

**Farihan Barghouti**

Jordan

**Arunodaya Barman**

Malaysia

**Rebecca Barr**

UK

**Elizabeth Barrett-Connor**

USA

**Hyam Bashour**

Syria

**Partha Basu**

India

**Majed Bata**

Jordan

**Moses Bateganya**

Uganda

**Ronald Battt**

USA

**Wafa Bawab**

Lebanon

**Angela Bazzi**

USA

**Aris Bechlioulis**

Greece

**Bei Bei**

Australia

**Ruud Bekkers**

Netherlands

**Tefera Belachew**

Ethiopia

**Laura Benaglia**

Italy

**Elizabeth Bertone-Johnson**

USA

**Jane Bertrand**

USA

**Rebecca Beyda**

USA

**Premananda Bharati**

India

**Neerja Bhatla**

India

**Nazan Bilgel**

Turkey

**Lorena Binfa**

Chile

**Kirsten Black**

Australia

**Tina Bloom**

USA

**Eric Blyth**

UK

**Daniel Bogale**

Ethiopia

**Jesper Bonde**

Denmark

**Matteo Bonzini**

Italy

**Remko Bosgraaf**

Netherlands

**Massimo Bracci**

Italy

**Daniel Breitkopf**

USA

**Molly Brewer**

USA

**Hilary Brown**

Canada

**Katherine Brown**

UK

**Joelle Brown**

USA

**Lauro Bucchi**

Italy

**Carol Bugge**

UK

**Thanh Bui**

Viet Nam

**Ann Burchell**

Canada

**Henry Burger**

Australia

**Helene Caillon**

France

**Elyce Cardonick**

USA

**Mark Carey**

Canada

**Marcia Carneiro**

Brazil

**Pilar Carrasco-Garrido**

Spain

**Rosario Castaño**

Spain

**Alejandra Castanon**

UK

**Patrick Catoire**

France

**Mario Cavagna**

USA

**Christina Chambers**

USA

**Arundhati Char**

India

**Dongbao Chen**

USA

**Donna Chizen**

Canada

**Donna Chung**

Australia

**Crystal Clark**

USA

**Kelly Cobey**

Canada

**Richard Cone**

USA

**Xavier Corbella Virós**

Spain

**Ali Kagan Coskun**

Turkey

**Jeremy Couper**

Australia

**Annie Cushing**

UK

**Michele Da Broi**

Brazil

**Rada Dagher**

USA

**Stefania D'Angelo**

UK

**Chantal Daumerie**

Belgium

**Melissa A. Davey-Rothwell**

USA

**Matthias David**

Germany

**Karen Davies**

UK

**Ayesha De Costa**

Sweden

**Mary De Silva**

UK

**Amanda Deeks**

Australia

**Thérèse Delvaux**

Benin

**Lynette Denny**

South Africa

**Haryana Dhillon**

Australia

**Cyril Dim**

Nigeria

**Mai Do**

USA

**Yusuf Cetin Doganer**

Turkey

**Wybo Dondorp**

Netherlands

**Tam Truong Donnelly**

Canada

**Frances Doran**

Australia

**Alison Drake**

USA

**Kristin Dunkle**

USA

**Christine East**

Australia

**Gillian Eastgate**

Australia

**Gillian Einstein**

Canada

**Sebastian Eliason**

Ghana

**Khalifa Elmusharaf**

Sudan

**Karen Ertel**

USA

**Cam Escoffery**

USA

**Adrienne Ettinger**

USA

**Euzebus Ezugwu**

Nigeria

**Alexandre Faisal-Cury**

Brazil

**Jamie Feldman**

USA

**Deborah Fenlon**

UK

**Bradford Fenton**

USA

**Herve Fernandez**

France

**Tesfaye Regassa Feyissa**

Ethiopia

**Mary Fisher**

USA

**Arthur Fleischer**

USA

**Andrew Flower**

UK

**Randi Foraker**

USA

**Caren Frost**

USA

**Franco Fulciniti**

Italy

**Qinglei Gao**

China

**Hongshan Ge**

China

**Caitlin Gerdts**

USA

**Paolo Giorgi Rossi**

Italy

**Nancy Glass**

USA

**Jeannine Goetz**

USA

**John Gohagan**

USA

**Mara Goldman**

USA

**Audra Gollenberg**

USA

**Brett Gordon**

Australia

**Shelley Gorman**

Australia

**Rebecca Grainger**

New Zealand

**Catherine Granger**

Australia

**Violanda Grigorescu**

USA

**Marika Guggisberg**

Australia

**Solomon K Gumanga**

Ghana

**Lisa Haddad**

USA

**Maria Hadjifrangiskou**

USA

**Helen Haines**

Australia

**Vivienne Hanley**

UK

**Estrid Stæhr Hansen**

Denmark

**Lisa Hanson**

USA

**Diane Harper**

USA

**Melissa Harris**

Australia

**Laura Harris**

USA

**Emily Harville**

USA

**Abigail Hatcher**

South Africa

**Kathy Hegadoren**

Canada

**Ingrid Hellstrom**

Sweden

**Frans Helmerhorst**

Netherlands

**Maggie Hendry**

UK

**Bernardo Hernandez**

USA

**Wendy Heywood**

Australia

**Andrew Hill**

New Zealand

**Grace Hillyer**

USA

**Janet Hirst**

UK

**Kelsey Holt**

USA

**Matthew Hopkins**

USA

**Reihaneh Hosseini**

Iran

**Louise Michele Howard**

UK

**Ming-I Hsu**

Taiwan

**Vivian Huang**

Canada

**Megan Huchko**

USA

**Myra Hunter**

UK

**Thelma Hurd**

USA

**Sanusi Ibrahim**

Nigeria

**Nneka Iloanusi**

Nigeria

**Ugo Indraccolo**

Italy

**Mohamad Irani**

USA

**Mercy Isichei**

Nigeria

**Rafiqul Islam**

Bangladesh

**Lisa Iversen**

UK

**Nadia Jahangiri**

Iran

**D. Elizabeth Jesse**

USA

**Nattinee Jitnarin**

USA

**Caroline Jones**

UK

**Heidi Jones**

USA

**Esmè Jordaan**

South Africa

**Deborah Karasek**

USA

**Rajendra Karkee**

Nepal

**Teketo Kassaw**

Ethiopia

**Cihan Kaya**

Turkey

**Nkomba Kayeyi**

Norway

**Sarah Keadle**

USA

**Deanna Kepka**

USA

**Zeinab Khadr**

Egypt

**Anne Khasakhala**

Kenya

**Celso Khosa**

Mozambique

**Fanny Kilpi**

Finland

**Min Jung Kim**

South Korea

**Evan Kleiman**

USA

**M. Tish Knobf**

USA

**Yi-Li Ko**

Taiwan

**Nikolaos Kofotolis**

Greece

**Jung-Nien Lai**

China

**Rebecca Landy**

UK

**Katrin Lang**

Estonia

**Gerri Lasiuk**

Canada

**Pallavi Latthe**

UK

**Justin Lavner**

USA

**Angela Lawson**

USA

**Huynh-Nhu Le**

USA

**Deborah Legorreta**

Mexico

**Jason Leider**

USA

**Charlotte Lenselink**

Netherlands

**Iñaki Lete**

Spain

**Lucy Lewis**

Australia

**Sonia Lewycka**

New Zealand

**Els Leye**

Belgium

**Chung-Yi Li**

Taiwan

**Michal Liebergall**

Israel

**Xiong Likuan**

China

**Jinn Lin**

Taiwan

**Annika Lindskog**

Sweden

**Robert Liston**

Canada

**Farah Lone**

Poland

**Virginia Lope**

Spain

**Artur Ludwin**

Poland

**Kazuyo Machiyama**

UK

**Margaret Madeleine**

USA

**Stéphanie Madill**

Canada

**Brianna Magnusson**

USA

**Michael Johnson Mahande**

Tanzania

**Pranitha Maharaj**

South Africa

**Mats Malqvist**

Sweden

**Suzanne Maman**

USA

**Michele Manahan**

USA

**Vinoj Manning**

India

**Roberto Marci**

Italy

**Desalegn Markos**

Ethiopia

**Morgan Marks**

USA

**Victoria Martinez Moron**

Spain

**Koji Matsuo**

USA

**Maria Matteo**

Italy

**Dimitrios Mavrelos**

UK

**Michael Mbizvo**

Switzerland

**Janet Mccabe**

Canada

**Angele Mcgrady**

USA

**Ian Mclennan**

New Zealand

**Bindu Mehta-Chimote**

India

**Andrea Mejia Rios**

Colombia

**Nicolas Mendoza**

Spain

**Melissa A. Merritt**

UK

**Katrina Milaney**

Canada

**Andrea Milbourne**

USA

**Karen Miller**

USA

**Silvia Minozzi**

Italy

**Suneeta Mittal**

India

**Bente Moen**

Norway

**Mitike Molla**

Ethiopia

**Patricia Moorman**

USA

**Kathryn Moracco**

USA

**Jane Morrell**

UK

**Morten Moshagen**

Germany

**Zulf Mughal**

UK

**Tamire Mulugeta**

Ethiopia

**Matthew Mulvey**

USA

**Namuunda Mutombo**

Kenya

**Ludovico Muzii**

Italy

**Shizar Nahidi**

Australia

**Aziza Nassar**

USA

**Joseph Nassif**

Lebanon

**Binod Neupane**

Canada

**Louise Newman**

Australia

**José Luis Neyro**

Spain

**Ernest Ng**

Hong Kong

**Enock Ngome**

Botswana

**Tram Nguyen**

Australia

**Hanh Nguyen**

Viet Nam

**Virginie Nicaise**

France

**Edgar Nieto**

Venezuela

**Maryam Niknejadi**

Iran

**Zeljka Nikolic**

Serbia

**Abigail Norris Turner**

USA

**Krzysztof Nowosielski**

Poland

**Theophilus Nwankwo**

Nigeria

**Theo Nwankwo**

Nigeria

**Samuel Obi**

Nigeria

**Shawn O'Connor**

Canada

**Björn Oddens**

Netherlands

**Kirsten Oinonen**

Canada

**Chinelo Okigbo**

Nigeria

**Ellinor Olander**

UK

**Margaret Olsen**

USA

**Suzanne O'Neill**

USA

**Maricianah Atieno Onono**

Kenya

**Livia Elisa Ortensi**

Italy

**Emre Pabuccu**

Turkey

**Manoranjan Pal**

India

**Santiago Palacios**

Spain

**Parisa Parsa**

Iran

**Harnish Patel**

UK

**Freda Patterson**

USA

**Mandira Paul**

Sweden

**Patricia Pautier**

France

**Tao Peng**

China

**Carolyn Pepper**

USA

**Robert L. Peralta**

USA

**Kath Peters**

Australia

**Patrick Petignat**

Switzerland

**Stamatios Petousis**

Greece

**Karen Phillips**

Canada

**Jashvant Poeran**

USA

**Omero Poli-Neto**

Brazil

**Ricki Pollycove**

USA

**Nancy Poole**

Canada

**Maria Grazia Porpora**

Italy

**Oriol Porta**

Spain

**Jerilynn Prior**

Canada

**Erica Prussing**

USA

**Carol Pullen**

USA

**Alexandra Purdue-Smithe**

USA

**Grzegorz Raba**

Poland

**Maya Ragavan**

USA

**Lauren Ralph**

USA

**Nancy Reame**

USA

**David Reardon**

USA

**Ronald Regal**

USA

**Jennifer Reingle**

USA

**Philip Robinson**

Australia

**Marta Rondon**

Peru

**Dana Roque**

USA

**Guri Rortveit**

Norway

**Sue Ross**

Canada

**Alberto Rossi**

Italy

**Ingrid Rowlands**

Australia

**Chiara Ruini**

Italy

**Joseph Ruminjo**

USA

**Nancy Russo**

USA

**Gideon Rutaremwa**

Ethiopia

**Riadh Sadik**

Sweden

**Gianni Saguatti**

Italy

**Daniel Salica**

Spain

**Tarik Sammour**

Australia

**Ariel Sanchez**

Argentina

**Maureen Sanderson**

USA

**Pavlos Sarafis**

Greece

**Ertan Saridogan**

UK

**David Sarwer**

USA

**Donald Saucier**

USA

**Takudzwa Sayi**

USA

**Karen Scanlon**

UK

**Channa Schmeink**

Netherlands

**Peter Schwartz**

USA

**Rebecca Sear**

UK

**Katarina Sedlecky**

Serbia

**Laura Serrant**

UK

**Omar Shaaban**

Egypt

**Donat Shamba**

Tanzania

**Larissa Shamseer**

Canada

**Mridula Shankar**

Australia

**Janet Shaw**

USA

**Jill Shawe**

UK

**Estelle Sidze**

Kenya

**Marushka L Silveira**

USA

**Makeda Sinaga**

Ethiopia

**Selina Smith**

USA

**Mickey Sperlich**

USA

**Juliane Spiegler**

Germany

**Sheila Sprague**

Canada

**Flaminio Squazzoni**

Israel

**Lesley Stafford**

Australia

**Alana Steffen**

USA

**Elizabeth Stier**

USA

**Jamila Stockman**

USA

**Beth Sundstrom**

USA

**Vanphanom Sychareun**

Laos

**Jennifer Tabler**

USA

**Hiroshi Tamura**

Japan

**Bee Kang Tan**

UK

**Cara Tannenbaum**

Canada

**Meredith Tavener**

Australia

**Diana Taylor**

USA

**Parisa Tehranifar**

USA

**Louise Terry**

UK

**Doreth Teunissen**

Netherlands

**Harshad Thakur**

India

**Ravi Thiara**

UK

**David Thomas**

USA

**Lisa Thompson**

USA

**Gill Thomson**

UK

**Tanya Timeva**

Bulgaria

**Angela Todd**

Australia

**Elena Toffol**

Finland

**Giovanni A Tommaselli**

Italy

**Adetunji Toriola**

USA

**Ha Trinh**

USA

**Karen Troy**

USA

**Lisa Troy**

USA

**Jennifer Tsai**

USA

**Sibil Tschudin**

Switzerland

**Katherine Tumlinson**

USA

**Uche Umeh**

Nigeria

**M. Renee Umstattd Meyer**

USA

**Ushma Upadhyay**

USA

**Lianne Urada**

USA

**Sameer Valsangkar**

India

**Sabine Van Den Akker**

Netherlands

**Olga Van Den Akker**

UK

**Emer Van Ryswyk**

Australia

**Douwe Verkuyl**

Netherlands

**Giovanni Veronesi**

Italy

**Lakshmi Vijayakumar**

India

**Amanda Vincent**

Australia

**Lorraine Walker**

USA

**Lewis Wall**

USA

**Sophie Warembourg**

France

**Charlotte Warren**

Kenya

**Melissa Watt**

USA

**Lori Weeks**

Canada

**Ram Weiss**

Israel

**Eliud Wekesa**

Kenya

**Benedict Weobong**

Ghana

**Sandra West**

Australia

**Nicole Westmarland**

UK

**Britta Wigginton**

Australia

**Mandika Wijeyaratne**

Sri Lanka

**Lesley Wilkes**

Australia

**Mark Willems**

UK

**Emma Williams**

USA

**Mellissa Withers**

USA

**Penny Wright**

UK

**Chi-Jung Wu**

Taiwan

**Hongwei Xu**

USA

**Kapil Yadav**

India

**Teymoor Yary**

Iran

**Paul Yong**

Canada

**Nese Yuksel**

Canada

**Sofija Zagarins**

USA

**Deirdre Zander-Fox**

Australia

**Chengquan Zhao**

USA

**Guangyu Zhou**

Germany

